# O03 Scale and spread of a quality improvement initiative promoting metronidazole IV to oral switch (IVOST) in the acute hospital setting: antimicrobial stewardship, patient safety, workforce and environmental sustainability benefits

**DOI:** 10.1093/jacamr/dlae136.003

**Published:** 2024-08-23

**Authors:** R Rodger, A Robertson, E Thompson, K Downie, S Thompson, G Ray, L Chisholm, R Hillson

**Affiliations:** NHS Greater Glasgow & Clyde, Glasgow, UK; NHS Greater Glasgow & Clyde, Glasgow, UK; NHS Greater Glasgow & Clyde, Glasgow, UK; NHS Greater Glasgow & Clyde, Glasgow, UK; NHS Greater Glasgow & Clyde, Glasgow, UK; NHS Greater Glasgow & Clyde, Glasgow, UK; NHS Greater Glasgow & Clyde, Glasgow, UK; Centre for Sustainable Healthcare, Oxford, UK

## Abstract

**Background:**

In-hospital promotion of the oral route and IV to oral switch (IVOST)^1^ are key antimicrobial stewardship (AS) initiatives providing benefits for patients, staff and the environment, including reduced risk of line infections, medicine costs, nursing workload and plastic waste.^1,2^ A recent quality improvement (QI) initiative highlighting the environmental sustainability benefits of switching to oral metronidazole (bioavailability >90%) in surgical wards at the Royal Alexandra Hospital (RAH) resulted in a 45% median reduction in IV administrations.^3^

**Objectives:**

To scale and spread this QI initiative to all inpatient wards in NHSGGC Clyde hospitals (RAH, Inverclyde Royal and Vale of Leven) and measure the impact on AS, drug costs, nursing workload and environmental sustainability.

**Methods:**

A QI scale and spread approach was used including: targeted pharmacist/pharmacy technician prospective audit and feedback; presentations to medical, nursing and pharmacy teams; eye-catching posters on wards/electronic guideline platform; staff champions; and HEPMA IVOST prompts when prescribing or administering IV metronidazole.^4^ Oral and IV metronidazole usage data was calculated at baseline and for 36 months post-change. Benefits in terms of reduced IV administrations, drug costs and nursing time saved^5^ were calculated. Plastic waste reduction (giving sets, single use plastic containers, cannula, safety needles, gloves and aprons) associated with switching from IV to oral metronidazole was calculated in terms of carbon dioxide equivalent (CO_2_e) emissions using a hybrid carbon footprinting methodology and emissions factor databases.^6,7^

**Results:**

There was a 64% median reduction in IV metronidazole defined daily doses (DDDs) (Figure 1) equating to 1977 and 23 724 fewer IV metronidazole administrations monthly and annually, respectively. The equivalent nursing time saved was 659 h per month and 7908 h per year. There was a 26% median shift in percentage IV metronidazole of total (IV plus oral) use (Figure 2). There was a 38% median reduction in metronidazole drug cost (Figure 3). The carbon footprint saving achieved from switching from IV to oral metronidazole was calculated as 1.48111 kg CO_2_e per dose, equating to 2928 kg CO_2_e per month and an annual carbon footprint saving of 35.1 tonnes CO_2_e; refer to Table 1 for equivalents.

**Conclusions:**

A QI scale and spread approach to raise awareness of the multidisciplinary team to the benefits of appropriate switch from IV to oral metronidazole resulted in a change in prescribing behaviour and a significant reduction in IV administrations. This is important in terms of improved AS, patient safety, workforce cost and efficiency and environmental sustainability.

**Figure 1.**
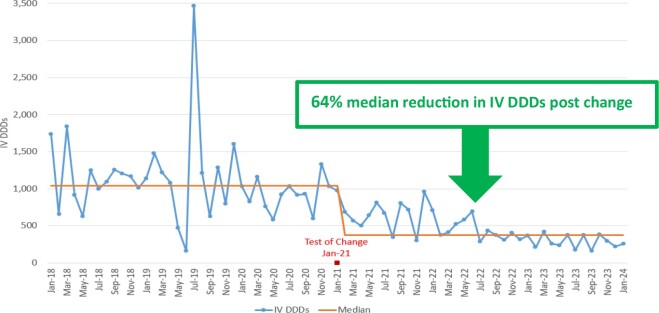
Clyde metronidazole IV DDDs.

**Figure 2.**
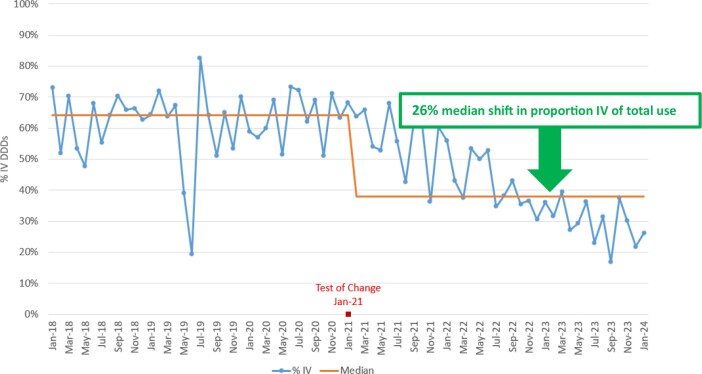
Clyde metronidazole percentage IV DDDs of oral + IV DDDs.

**Figure 3.**
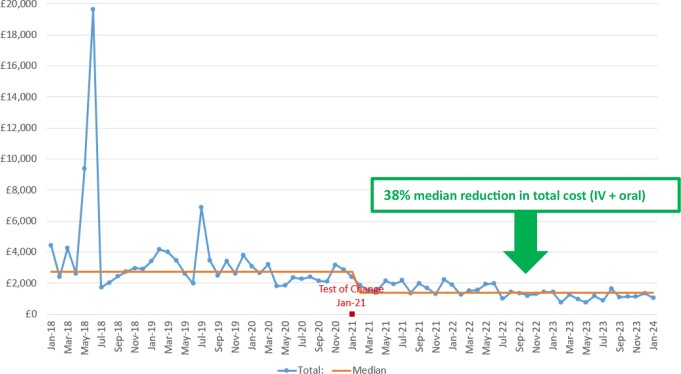
Clyde metronidazole total IV + oral spend.

**Table 1.** Carbon dioxide equivalents

Procedure	kg CO_2_e per unit	Units equivalent to 35.1 tonnes CO_2_e per year
IV to oral metronidazole switch	1.48111	23 724
1 cataract surgery in the UK	180	195
1 ambulance journey (single trip)	36.1	972
1 GP appointment	6	5850
1 mile in an average-sized car	0.28306	124 001.98 miles or driving around the world 5 times
